# The clinical value of combined assessment of substantia nigra morphology and intracranial hemodynamics via transcranial ultrasound in the diagnosis and differentiation of Parkinson’s disease

**DOI:** 10.3389/fneur.2026.1913166

**Published:** 2026-09-24

**Authors:** Liping Liang, Qiaotian Cui

**Affiliations:** Department of Ultrasound, The Second Affiliated Hospital of Baotou Medical College, Inner Mongolia University of Science and Technology, Baotou, Inner Mongolia, China

**Keywords:** diagnosis, hemodynamics, Parkinson’s disease, substantia nigra hyperechogenicity, transcranial sonography

## Abstract

**Background:**

Parkinson’s disease (PD) is a common neurodegenerative disease, and its clinical differentiation from Parkinson-plus syndromes (PPS) remains challenging due to the lack of highly specific biomarkers. Although dopamine transporter single-photon emission computed tomography (DAT-SPECT) provides objective evidence of dopaminergic denervation, its limited value in distinguishing PD from PPS and restricted availability underscore the need for accessible and reliable diagnostic tools. This study aimed to evaluate a novel, non-invasive, and convenient auxiliary diagnostic approach for the diagnosis and differentiation of PD by integrating the assessment of substantia nigra hyperechogenicity (SN+) and intracranial hemodynamics using transcranial ultrasound.

**Methods:**

A total of 68 PD patients (PD group), 30 PPS patients (PPS group), and 80 healthy controls (NC group) were enrolled between June 2023 and December 2025. All subjects underwent Transcranial Sonography (TCS) to measure the area of SN+ and Transcranial Color Doppler (TCCD) to measure intracranial vascular hemodynamic parameters. Intergroup differences were analyzed, and receiver operating characteristic (ROC) curves were used to evaluate the diagnostic efficacy of individual parameters and their combination.

**Results:**

Ultrasound parameters of the substantia nigra, including unilateral and bilateral hyperechogenicity, were markedly elevated in PD patients compared to PPS and control groups (*p* < 0.05). Additionally, the PD group exhibited altered hemodynamic profiles, with significant differences in resistance index and diastolic flow velocity of the middle cerebral artery (MCA Vd) relative to controls. ROC curve analyses indicated that the diagnostic accuracy for PD using the SN+ area was high (AUC = 0.918), while a combined model incorporating TCS and TCCD parameters achieved a superior AUC of 0.945, with a sensitivity of 91.2% and specificity of 95.0%.

**Conclusion:**

TCS-detected SN+ is an effective imaging biomarker for PD. Combining SN+ with MCA Vd demonstrated superior diagnostic efficacy (AUC = 0.945) compared with either parameter alone, providing a non-invasive multimodality tool for distinguishing PD from non-PD individuals (including PPS and healthy controls).

## 1 Introduction

Parkinson’s disease (PD), as a progressive neurodegenerative disorder, is pathologically characterized by the progressive depletion of dopaminergic neurons in the substantia nigra pars compacta of the midbrain and the pathological accumulation of Lewy bodies. The clinical phenotype not only includes typical motor dysfunctions such as bradykinesia, resting tremor, muscle rigidity, and postural balance disorders, but is also frequently associated with various non-motor symptoms. With accelerating global population aging, the incidence of PD has significantly increased, which not only exacerbates the caregiving burden on patients’ families but also poses severe economic challenges to the public health system (1, 2). Although current clinical diagnosis primarily adheres to the International Movement Disorder Society (MDS) clinical diagnostic criteria (3), accurate differentiation based solely on clinical symptoms remains difficult in the early stages. This is due to the lack of symptom specificity and significant phenotypic overlap with Parkinson-plus syndromes (PPS), such as progressive supranuclear palsy (PSP) and multiple system atrophy (MSA). This diagnostic uncertainty often leads to delayed or inappropriate treatment, thereby affecting patient prognosis and quality of life. Although DAT-SPECT can provide objective evidence of dopaminergic denervation, its limited value in distinguishing PD from atypical parkinsonian syndromes such as PSP or MSA, and its often restricted availability, mean that highly specific and reliable objective biomarkers are still urgently needed for early differential diagnosis. Therefore, there is an urgent clinical need for a non-invasive, convenient, and highly specific objective biological marker to improve the accuracy of early PD diagnosis and achieve refined differentiation among various Parkinsonian syndrome subtypes.

In addition to the characteristic MRI signs of Parkinson’s disease and atypical parkinsonism (4), TCS has garnered significant attention for its ability to penetrate the temporal window and clearly visualize the brain parenchyma structure (5–8). Multiple previous studies, including a systematic review and meta-analysis (9), have indicated that transcranial ultrasound-detected hyperechogenicity of the substantia nigra (SN+) is a biomarker for Parkinson’s disease, which can be used to differentiate Parkinson’s disease from essential tremor and healthy controls, demonstrating high diagnostic sensitivity and specificity (5–7, 10). Furthermore, quantitative assessment of the SN+ area not only aids in differential diagnosis but also correlates closely with the risk of levodopa-induced dyskinesia (LID), suggesting its potential utility in predicting LID (11). However, although unilateral or bilateral SN+ is a typical feature of PD, similar substantia nigra echogenicity changes, albeit with significantly lower incidence rates, can be observed in some patients with atypical Parkinsonian syndromes (12), indicating that a single structural indicator remains limited in the differential diagnosis of complex cases. This underscores the need for complementary markers that reflect distinct facets of PD pathophysiology.

Beyond changes in brain parenchyma structure, the role of cerebrovascular functional status in the pathophysiological mechanisms of neurodegenerative diseases is increasingly prominent (13, 14). TCCD can non-invasively assess hemodynamic parameters of major intracranial arteries, such as the pulsatility index (PI), resistance index (RI), and blood flow velocity. These parameters reflect distal vascular bed resistance and cerebrovascular regulatory function (15). While cerebrovascular reactivity and flow velocity changes have been reported in PD patients with cardiovascular risk factors (16), increasing evidence suggests that endothelial dysfunction and impaired neurovascular coupling are intrinsic to PD pathophysiology, independent of traditional vascular risk factors (17). For instance, advanced MRI and transcranial Doppler studies have shown altered resting-state cerebral blood flow and reduced neurovascular coupling in PD patients without significant large-vessel disease (18). These findings suggest hemodynamic abnormalities are an inherent feature of parkinsonian disorders, not merely a consequence of comorbidity.

Although existing literature discusses the independent value of TCS for assessing substantia nigra structure (5–8) and TCCD for evaluating cerebrovascular function (13, 14, 19), research integrating these approaches remains scarce. Previous studies have examined SN+ alone (5, 9, 20) or hemodynamic parameters separately (14, 19, 21), and despite a systematic review (22) and earlier combined attempts in selected subgroups [e.g., Tsai et al. (23)], no study, to our knowledge, has systematically quantified both SN+ area and MCA Vd across PD, PPS, and healthy controls (after excluding severe cerebrovascular disease), nor explored their intrinsic correlation or complementarity. Given that SN+ alone may miss patients with negative findings and hemodynamic parameters alone lack specificity, the present study aimed to fill this gap by evaluating a combined TCS + TCCD model.

Therefore, developing a multimodal ultrasound diagnostic model that includes both structural and functional dimensions is particularly necessary. Based on the hypothesis that a combined TCS and TCCD model would provide superior diagnostic accuracy for differentiating PD from non-PD individuals (including PPS and healthy controls) compared with either modality alone, this study aimed to evaluate the individual and combined diagnostic value of SN+ area and intracranial hemodynamic parameters, and to determine whether the combined model offers incremental diagnostic benefit over either parameter alone.

## 2 Methods

### 2.1 Research subjects

This study employed a cross-sectional design and recruited subjects from June 1, 2023, to December 31, 2025. Initially, a total of 78 PD patients, 36 PPS patients, and 91 healthy controls were recruited. Among them, 10 PD patients, 6 PPS patients, and 11 healthy controls were excluded due to inadequate bilateral temporal acoustic windows. Ultimately, 178 subjects were included, comprising 68 in the PD group, 30 in the PPS group, and 80 in the NC group. The three groups were matched at the group level for age and sex. This study was approved by the hospital ethics committee. The ethics review board of the Second Affiliated Hospital of Baotou Medical College approved this single-center study (Approval No: 2024-ZX053), and all participants provided written informed consent before enrollment.

Inclusion Criteria: ① All patients in the PD group were diagnosed according to the Movement Disorder Society (MDS) clinical diagnostic criteria for Parkinson’s disease (Postuma et al., 2015) (3). To facilitate standardized clinical application in the Chinese population, the Chinese Diagnostic Criteria for Parkinson’s Disease (2016 edition) (24)—which is the Chinese national adaptation of the MDS criteria—was also referenced for cross-checking, and no patient was included if they did not meet the MDS criteria. ② Patients in the PPS group were diagnosed with specific Parkinson-plus syndromes, including progressive supranuclear palsy (PSP), multiple system atrophy (MSA), dementia with Lewy bodies (DLB), and corticobasal syndrome (CBS), according to the respective international consensus diagnostic criteria for each disease (25–28).

Exclusion Criteria (applicable to both the PD and PPS groups): ① Presence of mental disorders; ② History of intracranial organic diseases such as brain injury or brain tumor; ③ Inadequate bilateral temporal acoustic windows; ④ Co-occurring systemic diseases that may affect hemodynamics, such as hypertension, hyperlipidemia, hyperglycemia, and coronary atherosclerosis. Additionally, patients in the PD group were required to be free of various secondary Parkinsonian syndromes, Parkinson-plus syndromes, and essential tremor; similarly, patients in the PPS group were required to be free of primary, secondary, and hereditary Parkinsonian syndromes.

Inclusion criteria for the healthy control group included: individuals who were healthy, matched by age and sex to the patient groups, capable of cooperating with the TCS examination, and possessed adequate temporal acoustic windows.

Exclusion criteria included: presence of comorbidities such as hypertension, hyperlipidemia, hyperglycemia, or coronary atherosclerosis; or a significant history of mental disorders, family history of PD, severe intracranial diseases, or prior intracranial surgery.

### 2.2 Collection of general clinical data

The age, gender, Systolic and diastolic blood pressure (SBP/DBP), education level, and smoking history of participants across the three groups were recorded. For PD and PPS patients, disease duration (years), levodopa equivalent daily dose (LEDD), UPDRS part III (motor score), and Hoehn & Yahr (H&Y) stage were assessed at enrollment. LEDD was calculated according to the Tomlinson conversion formula (29). For PD patients, H&Y stage was further categorized into <Stage III and ≥Stage III for subgroup analysis. These motor assessments (UPDRS III and H&Y stage) were not applicable to NC controls, as they were healthy individuals with no parkinsonian symptoms. All clinical data were collected at the time of enrollment. Table 1 summarizes the demographic and clinical characteristics of the three groups.

**Table 1:** Comparison of general information.

Variable	NC group (*N* = 80)	PD group (*N* = 68)	PPS group (*N* = 30)	*F*/*t*/*U*/*χ*^2^	*p*
Age^b^	67.70 ± 4.60	67.84 ± 4.66	66.97 ± 4.57	*0.724*	*0.696*
Gender^a^				*0.063*	*0.969*
Male	46 (57.50%)	40 (58.82%)	18 (60.00%)		
Female	34 (42.50%)	28 (41.18%)	12 (40.00%)		
Smoking History^a^	23 (28.75%)	20 (29.41%)	10 (33.33%)	*0.226*	*0.893*
Educational Attainment^a^				*0.280*	*1.000*
Illiterate	3 (3.75%)	2 (2.94%)	1 (3.33%)		
Primary School	16 (20.00%)	15 (22.06%)	6 (20.00%)		
Junior High School	35 (43.75%)	29 (42.65%)	14 (46.67%)		
High School and Above	26 (32.50%)	22 (32.35%)	9 (30.00%)		
SBP (mmHg)^b^	125.0 ± 14.0	128.5 ± 15.2	132.0 ± 16.8	*2.58*	*0.078*
DBP (mmHg)^b^	76.0 ± 9.5	78.5 ± 10.1	80.0 ± 11.5	*2.12*	*0.124*
Disease duration (years)^c^	–	4.5 ± 2.8	4.0 ± 2.5	*0.84*	*0.403*
LEDD (mg/d)^c^	–	526.4 ± 213.7	312.5 ± 186.2	*5.473*	*<0.001*
UPDRS III score ^c^	–	19.88 ± 7.76	21.35 ± 7.92	0.887	*0.376*
H&Ystage (median, IQR) ^d^	–	2.0 (1.5–3.0)	2.0 (1.5–3.0)	1,246.5	*0.441*
<Stage III	–	48 (70.59%)	–	–	–
≥Stage III	–	20 (29.41%)	–	–	–

a: Chi-square test; b: One-way ANOVA; c: Independent samples *t*-test (PD vs PPS); d: Mann–Whitney *U* (PD vs PPS). Levodopa-equivalent daily dose was calculated according to the Tomlinson conversion formula (29). Italic values indicate statistical values, including test statistics and *p*-values.

### 2.3 Ultrasonography protocol

Two specially trained ultrasonographers in transcranial ultrasound, who were blinded to the clinical diagnoses of the subjects, performed the examinations using a Vivid E95 color Doppler ultrasound diagnostic system (GE, USA) with an M5Sc-D cardiac probe (frequency: 1.5–4.0 MHz). All examinations were conducted within one week of the clinical assessment. Subjects were positioned supine and scanned through the temporal and occipital windows. Primary instrument settings: dynamic range 45–55 dB, penetration depth 14–16 cm. All data were measured three times consecutively, and the mean value was calculated.

#### 2.3.1 Transcranial sonography (TCS) examination

The probe was placed on the temporal window, with the ultrasound beam parallel to the orbitomeatal line, to acquire a clear axial midbrain section image (characterized by a “butterfly-shaped” hypoechoic structure surrounded by a hyperechoic basal cistern, see Figure 1a). The image quality was assessed based on the clear visibility of the contralateral skull outline. The image was then frozen, and the midbrain region and the substantia nigra (SN) hyperechoic region (defined as ≥ grade III echo intensity; see Figure 1b) were manually delineated and measured. Unilateral and bilateral SN hyperechoic areas, along with their ratios relative to the midbrain area (substantia nigra-to-midbrain ratio, S/M ratio), were calculated. Referring to previous studies (30), subjects with an SN hyperechoic (SN+) area ≥ 0.20 cm^2^ were defined as the PD SN+ subgroup.

**Figure 1:**
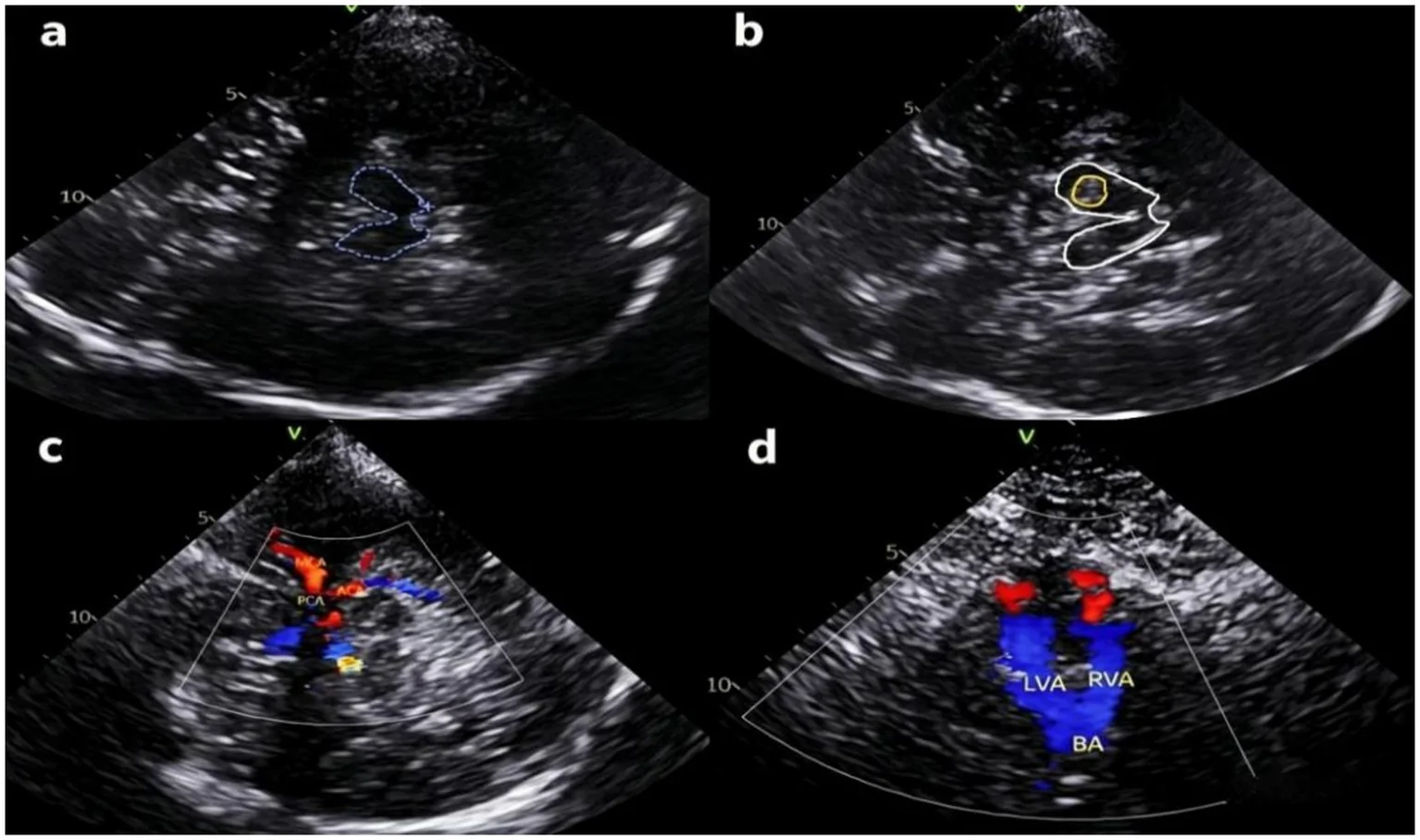
Transcranial midbrain substantia nigra ultrasound and hemodynamic imaging. **(a)** Normal axial view of the substantia nigra in the midbrain. The blue dotted line outlines the hypoechoic, butterfly-shaped midbrain structure, representing the standard imaging plane for transcranial parenchymal ultrasound. **(b)** An image showing increased echogenicity of the substantia nigra in a patient with Parkinson’s disease. The solid yellow line indicates the hyperechoic area of the substantia nigra (SN+), which is the manually outlined measurement region. **(c)** Color Doppler image of the midbrain plane obtained via the temporal window, demonstrating blood flow within the anterior circulation of the circle of Willis. MCA: Middle cerebral artery; ACA: Anterior cerebral artery; PCA: Posterior cerebral artery. **(d)** Color Doppler image of the vertebrobasilar arteries through the occipital window. LVA, Left vertebral artery; RVA, Right vertebral artery; BA, Basilar artery.

#### 2.3.2 Transcranial color doppler (TCCD) hemodynamic examination

① Temporal Window Examination: After obtaining the standard midbrain plane, the color Doppler mode was activated to visualize blood flow within the Circle of Willis (see Figure 1c). The middle cerebral artery (MCA), anterior cerebral artery (ACA), and posterior cerebral artery (PCA) were detected separately. Measurements were averaged over 3–5 consecutive stable cardiac cycles, Bilateral measurements for each artery were averaged for each participant, and the mean value was used for further analysis. To obtain the peak systolic velocity (*Vs*), end-diastolic velocity (Vd), pulsatility index (PI), and resistance index (RI). PI and RI were calculated as: PI = (*Vs* − Vd)/Vmean, and RI = (*Vs*–Vd) / Vs.

② Occipital Window Examination: The probe was placed between the posterior margin of the foramen magnum and the posterior margin of the first cervical vertebra spinous process, with the ultrasound beam directed toward the bridge of the nose, to acquire the blood flow spectra of the bilateral vertebral arteries (VA) and the basilar artery (BA) (see Figure 1d). The angle between the Doppler sampling line and the vessel axis was maintained at <60°. Using the same procedure as described for the temporal window, measurements were averaged over 3–5 consecutive stable cardiac cycles to obtain the *Vs*, Vd, PI, and RI.

### 2.4 Statistical methods

All statistical analyses were performed using SPSS 26.0 (IBM Corp., Armonk, NY, USA), except for the 10-fold cross-validation and related model performance computations, which were performed using R software (version 4.3.1; packages pROC and caret). Normality was assessed by the Shapiro–Wilk test. Normally distributed continuous variables are presented as mean ± SD; non-normally distributed variables as median (IQR).

For three-group comparisons (PD, PPS, NC), one-way ANOVA was used for normally distributed variables and the Kruskal–Wallis test for non-normally distributed variables. Each vessel segment (MCA, ACA, PCA, VA, BA) was compared independently across the three groups. Post-hoc pairwise comparisons were performed using Tukey’s HSD test (when variances were homogeneous) or Games–Howell test (when variances were unequal). For non-normally distributed data, Dunn’s test with Bonferroni correction was used. Within each family of related comparisons (e.g., substantia nigra parameters, or hemodynamic parameters for a given vessel segment), Bonferroni adjustment was applied to control Type I error.

To account for potential confounding, ANCOVA with age and sex as covariates was performed for the primary ultrasound parameters (SN+ area, MCA Vd, BA PI), with Bonferroni-corrected post-hoc comparisons.

UPDRS III and H&Y stage were compared between PD and PPS groups using appropriate tests (independent *t*-test or Mann–Whitney *U* test for UPDRS III; Mann–Whitney *U* test for H&Y). Categorical data were compared by *χ*^2^ test or Fisher’s exact test.

#### 2.4.1 Diagnostic model construction and validation

To evaluate the incremental diagnostic value of combining transcranial sonography (TCS) and transcranial color Doppler (TCCD) parameters, a binary logistic regression model was developed to discriminate PD patients from non-PD individuals (including PPS and healthy controls). The candidate predictors were substantia nigra hyperechogenicity (SN+) area and middle cerebral artery end-diastolic velocity (MCA Vd), which were selected *a priori* based on clinical plausibility and because they showed significant differences among the three groups in univariate analyses; they represent two complementary dimensions—nigral structural abnormality versus cerebral hemodynamic change. This choice also maintains clinical interpretability and avoids overfitting. The model equation was:


$$\text{logit}(p)=\beta_{0}+\beta ₁\times (\mathrm{SN}+\text{area})+\beta ₂\times (\mathrm{MCA}\;\mathrm{Vd})$$

where p is the predicted probability of Parkinson’s disease.

Model performance was assessed by the area under the receiver operating characteristic curve (AUC). ROC curves were constructed for SN + area, MCA Vd, and their combined logistic regression model. The optimal cutoff was determined by Youden’s index. AUCs were compared using DeLong’s test. To mitigate over-optimism, 10-fold cross-validation was performed using R software (version 4.3.1), and the cross-validated AUC was reported with its 95% confidence interval. A formal covariate-adjusted ROC analysis was not performed owing to the exploratory nature and limited sample size; the robustness of the findings was supported by the ANCOVA results and cross-validation. All diagnostic metrics, including optimal cutoffs, sensitivity, specificity, and AUC, were derived from the full study cohort. The 10-fold cross-validation was conducted solely to assess internal model stability and is reported separately.

A two-sided *p* < 0.05 was considered statistically significant.

## 3 Results

### 3.1 Comparison of general information

This study enrolled 178 subjects, including 68 PD patients, 30 PPS patients, and 80 healthy controls (NC). The three groups were well matched for age (*p* = 0.696) and sex distribution (*χ*^2^ = 0.063, *p* = 0.969). No significant differences were observed among the three groups in SBP, DBP, educational attainment, or smoking history (all *p* > 0.05). Among PD and PPS groups, disease duration, UPDRS III score, and H&Y stage showed no significant differences, whereas LEDD was significantly higher in the PD group (526.4 ± 213.7 vs. 312.5 ± 186.2 mg/d, *p* < 0.001). Given the potential influence of levodopa on cerebral hemodynamics, the higher LEDD in the PD group was considered in the interpretation of inter-group hemodynamic differences; however, as discussed, it is unlikely to fully explain the observed findings. PPS-specific severity scales (e.g., PSP Rating Scale, UMSARS) were not systematically extracted, which is acknowledged as a limitation. All demographic and clinical data are summarized in Table 1.

### 3.2 Comparison of midbrain substantia nigra among the three groups

The comparison of midbrain substantia nigra-related parameters among the three groups are presented in Table 2. The unilateral SN+ area, bilateral SN+ total area, and S/M ratio in the PD group were significantly higher than those in the PPS group and the NC group (all *p* < 0.05), whereas no significant differences were observed between the PPS and NC groups. After adjusting for age and sex via ANCOVA, the differences in unilateral SN+ area, bilateral SN+ total area, and S/M ratio among the three groups remained statistically significant (all adjusted *p* < 0.001). The adjusted means and 95% confidence intervals are presented in Supplementary Table S1. No statistically significant differences in midbrain area were found among the three groups. The SN+ positivity rate in the PD group (82.4%) was significantly higher than that in the PPS group (13.3%) and the NC group (11.3%).

**Table 2:** Comparison of Substantia Nigra-related parameters among the three groups of subjects.

Group	*N*	Unilateral SN+ area (cm^2^)	Bilateral SN+ total area (cm^2^)	Midbrain area (cm^2^)	*S*/*M* ratio (%)	SN+ positivity rate
PDgroup	68	0.28 ± 0.07^bc^	0.55 ± 0.05^bc^	4.80 ± 0.20	11.45 ± 0.93^bc^	82.4%^bc^
PPSgroup	30	0.11 ± 0.08^a^	0.18 ± 0.29^a^	4.80 ± 0.22	4.17 ± 0.65^a^	13.3%^a^
NCgroup	80	0.09 ± 0.07^a^	0.15 ± 0.03^a^	4.77 ± 0.21	3.14 ± 0.54^a^	11.3%^a^
*F*/*χ*^2^		*F = 86.758*	*F = 21.229*	*F = 1.287*	*F = 12.165*	*χ^2^ = 88.115*
*p*		<*0.01*	<*0.01*	*0.525*	<*0.01*	<*0.01*

a: *p* < 0.05 vs. PD group; b: *p* < 0.05 vs. PPS group; c: *p* < 0.05 vs. NC group. All PPS vs. NC comparisons were non-significant (*p* > 0.05), thus no c annotations appear in the table. Italic values indicate statistical values, including test statistics and *p*-values.

### 3.3 Comparison of hemodynamics among the three groups

Table 3 presents the comparison of intracranial artery hemodynamic parameters. The NC group showed significantly lower PI and RI in the MCA and BA, as well as lower RI in the VA, compared with both the PD and PPS groups (all *p* < 0.05). The VA PI was significantly lower in the NC group than in the PD group (*p* < 0.05), but did not differ significantly from the PPS group. Regarding flow velocities, the NC group showed higher MCA Vd than both PD and PPS groups (all *p* < 0.05), higher BA Vd than the PD group (*p* < 0.05), and higher PCA *Vs* than both PD and PPS groups (all *p* < 0.05). No significant intergroup differences were found in the ACA parameters. The PD group exhibited significantly higher PI values in the BA and VA compared with the PPS group (all *p* < 0.05). For the BA RI, both the PD and PPS groups demonstrated significantly higher values compared to the NC group (all *p* < 0.05), with no significant difference observed between the PD and PPS groups. After adjusting for age and sex via ANCOVA, the differences in MCA Vd and BA PI among groups remained statistically significant (all adjusted *p* < 0.05). The adjusted means and 95% confidence intervals are presented in Supplementary Table S1.

**Table 3:** Comparison of intracranial vascular hemodynamic parameters among the three groups of subjects (X ± s).

Artery	Parameter	NC group (*n* = 80)	PD group (*n* = 68)	PPS group (*n* = 30)
MCA	*Vs* (cm/s)	100.26 ± 7.25	99.17 ± 5.41	97.34 ± 6.03
	Vd (cm/s)	38.04 ± 4.55^ab^	35.44 ± 3.55^c^	35.60 ± 4.12^c^
	PI	0.70 ± 0.06^ab^	0.95 ± 0.08^c^	0.94 ± 0.09^c^
	RI	0.60 ± 0.08^ab^	0.71 ± 0.07^c^	0.70 ± 0.09^c^
ACA	*Vs* (cm/s)	80.28 ± 6.11	80.69 ± 5.44	80.68 ± 4.73
	Vd (cm/s)	34.39 ± 3.57	33.67 ± 3.25	34.20 ± 2.70
	PI	0.66 ± 0.06	0.78 ± 0.06	0.77 ± 0.05
	RI	0.66 ± 0.09	0.64 ± 0.07	0.65 ± 0.07
PCA	*Vs* (cm/s)	68.64 ± 3.29^ab^	65.58 ± 3.37^c^	66.84 ± 2.66^c^
	Vd (cm/s)	24.34 ± 3.20	23.37 ± 3.22	23.57 ± 3.01
	PI	0.75 ± 0.06	0.77 ± 0.08	0.74 ± 0.05
	RI	0.66 ± 0.08	0.63 ± 0.08	0.65 ± 0.08
VA	*Vs* (cm/s)	56.02 ± 2.94	54.39 ± 2.55	55.05 ± 2.84
	Vd (cm/s)	32.02 ± 1.96	31.04 ± 3.48	31.92 ± 2.28
	PI	0.73 ± 0.06^a^	0.81 ± 0.05^bc^	0.74 ± 0.05^a^
	RI	0.62 ± 0.07^ab^	0.72 ± 0.05^c^	0.74 ± 0.05^c^
BA	*Vs* (cm/s)	65.90 ± 4.27	67.09 ± 5.49	67.15 ± 4.24
	Vd (cm/s)	43.22 ± 3.05^a^	41.88 ± 2.51^c^	42.52 ± 2.78
	PI	0.67 ± 0.09^ab^	0.95 ± 0.04^bc^	0.87 ± 0.04^ac^
	RI	0.60 ± 0.04^ab^	0.74 ± 0.06^c^	0.72 ± 0.04^c^

a: *p* < 0.05 vs. PD group; b: *p* < 0.05 vs. PPS group; c: *p* < 0.05 vs. NC group. MCA, ACA, PCA, BA, and VA denote the middle cerebral artery, anterior cerebral artery, posterior cerebral artery, basilar artery, and vertebral artery, respectively. Abbreviations: *Vs*, Vd, PI, and RI represent systolic blood flow velocity, end-diastolic blood flow velocity, pulsatility index, and resistance index, respectively.

### 3.4 Diagnostic efficacy

A binary logistic regression model combining SN+ area and MCA Vd was constructed to discriminate PD from non-PD individuals (including PPS and healthy controls). All metrics in Table 4 were calculated on the full dataset; the 10-fold cross-validated AUC is reported separately for internal validation. The AUC for diagnosing PD using the unilateral SN+ area alone was 0.918 (optimal cutoff: 0.24 cm^2^), whereas the MCA Vd alone yielded an AUC of 0.665 (optimal cutoff: 39.26 cm/s). Combining both parameters improved the AUC to 0.945, with a sensitivity of 91.2% and a specificity of 95.0%. DeLong’s test showed that the combined model was significantly superior to either parameter alone (both *p* < 0.05). Ten-fold cross-validation confirmed good model stability (AUC = 0.915). Full statistical details are provided in Table 4 and Figure 2.

**Table 4:** Analysis of diagnostic performance of transcranial ultrasound.

Diagnostic indicator	Cut-off value	Sensitivity (%)	Specificity (%)	AUC	95%CI	AUC *p* value^(a)^
Unilateral SN+ area	0.24 cm^2^	82.3	98.6	0.918	0.871–0.965	*0.002*
MCA Vd	39.26 cm/s	91.0	41.2	0.665	0.578–0.753	*0.003*
Combination of both	–	91.2	95.0	0.945	0.907–0.984	*0.002*

^(a)^*p* values test the null hypothesis that AUC = 0.5. DeLong’s test: combined model vs. unilateral SN+ area, *Z* = 2.12, *p* = 0.034; combined model vs. MCA Vd, *Z* = 5.31, *p* < 0.001. All metrics are apparent (training-set) values; the 10-fold cross-validated AUC of the combined model was 0.915 (95% CI: 0.880–0.950). Italic values indicate statistical values, including test statistics and *p*-values.

**Figure 2:**
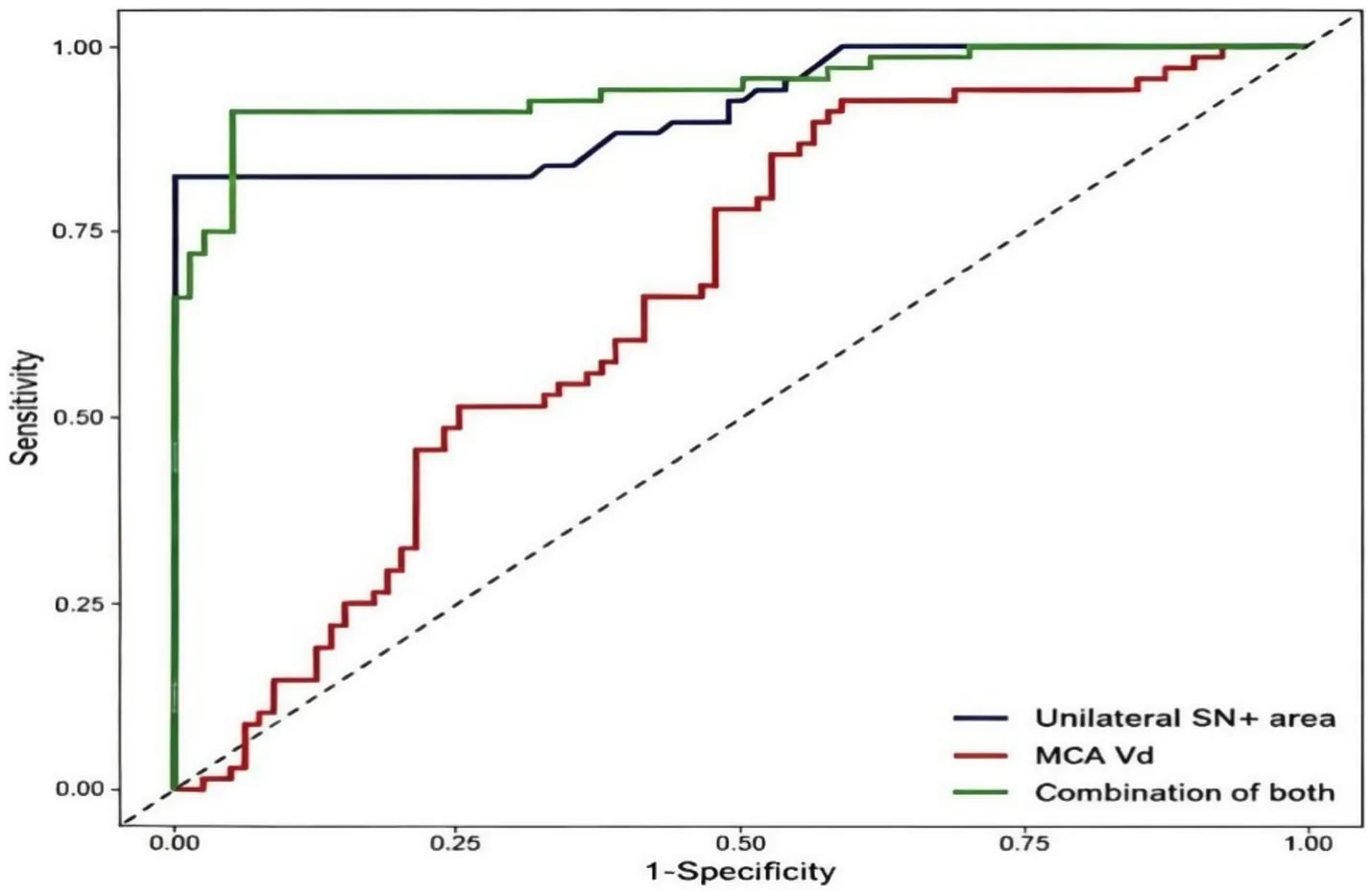
Diagnostic efficacy of combined assessment of substantia nigra echogenicity and cerebral hemodynamic parameters using transcranial ultrasound for Parkinson’s disease (PD).

## 4 Discussion

This study demonstrated that combining the SN+ area and MCA Vd significantly improved the differentiation of PD from non-PD participants (AUC = 0.945). This supports the synergistic benefit of integrating structural (TCS) and functional (TCCD) ultrasound parameters in a single, non-invasive examination, and suggests a clinically feasible approach that overcomes the limitations inherent to single-parameter strategies.

### 4.1 The value of substantia nigra Hyperechogenicity in the differential diagnosis of PD

In this study, the substantia nigra hyperechogenicity area and the S/M ratio were significantly higher in PD patients than in NC and PPS patients, while no significant difference was observed between the PPS and NC groups. This is consistent with previous reports (31–33). However, because 10–15% of healthy individuals may also exhibit SN+ (34), this marker likely reflects a susceptibility state rather than an absolute basis for a definitive PD diagnosis. It should therefore be interpreted together with other indicators, especially when SN+ is equivocal or absent; in such cases, complementary functional parameters—such as TCCD-derived hemodynamic indices—may provide additional diagnostic information and reduce reliance on a single structural marker.

### 4.2 Cerebral hemodynamic changes in PD patients and their significance

Both PD and PPS patients showed significantly reduced diastolic flow velocities in the MCA and basilar artery, together with increased RI and PI, suggesting impaired cerebrovascular regulatory mechanisms or microcirculatory disturbances shared by PD and related disorders (35). Notably, the PI of the vertebrobasilar system was significantly higher in the PD group than in the PPS group and healthy controls, indicating a unique high-resistance state in PD. Although previous literature reported a positive correlation between carotid artery stenosis and intracranial PI (36), this explanation is unlikely in our cohort because patients with large-vessel stenosis were excluded. The elevated PI may therefore reflect a combination of increased distal vascular resistance, impaired cerebrovascular autoregulation, and autonomic dysfunction commonly seen in PD (37). In contrast to acute hemodynamic changes (e.g., increased ipsilateral MCA velocity with elevated PI after stenting) or the relatively stable PI observed in healthy elderly individuals after aerobic exercise (38), the persistent high-resistance state in our PD patients suggests a chronic pathological process rather than an acute physiological fluctuation. Moreover, the fact that the high-resistance pattern was most evident during the diastolic phase may link it to impaired cardiac diastolic function or autonomic dysfunction (39–41). Further studies using more direct measures of microvascular integrity (e.g., MRI-based techniques) are needed to clarify the underlying mechanisms.

Therefore, high vertebrobasilar artery resistance (reflected by elevated PI in the VA and BA) may serve as a potential hemodynamic marker for distinguishing PD from other parkinsonian syndromes. Apart from these resistance parameters, no significant differences were found in other TCCD hemodynamic parameters between the PD and PPS groups, suggesting that the value of individual TCCD parameters in differential diagnosis remains limited. Additionally, although the MCA Vd could not directly differentiate PD from PPS, its combination with the SN+ area significantly improved diagnostic performance. Future studies with larger sample sizes and refined PPS subtype classification are warranted to validate these findings.

Levodopa may exert vasodilatory properties and modulate regional cerebral perfusion in PD (42), raising the possibility that dopaminergic medication confounds transcranial ultrasound-derived hemodynamic measurements. In our cohort, the levodopa-equivalent daily dose (LEDD) was significantly higher in the PD group than in the PPS group. If levodopa substantially altered cerebral blood-flow velocity, PD patients would be expected to show marked medication-related hemodynamic changes. However, previous work suggests that levodopa-mediated vascular influence is region-specific rather than producing a consistent global alteration of cerebral blood-flow velocity detectable by transcranial ultrasound (43). Although many PPS patients also receive levodopa as a therapeutic trial, their motor response is generally poor, resulting in a lower average LEDD. Given these baseline characteristics and published evidence, we cannot completely rule out partial vasodilatory interference from levodopa, but it is unlikely to fully account for the intergroup hemodynamic differences observed between PD and PPS. Future prospective studies with strictly standardized medication status are required to clarify this issue.

### 4.3 Synergistic advantages of combined diagnosis

A key finding of this study is that combining TCS (a structural indicator) and TCCD (a functional indicator) significantly improved diagnostic efficacy for PD. Using the SN+ area alone, while having high specificity (98.6%), had relatively limited sensitivity (82.3%). Using the MCA Vd alone, while having high sensitivity (91.0%), had lower specificity (41.2%). When combined, these indicators maintained high sensitivity (91.2%) while substantially increasing specificity to 95.0%.

This fully demonstrates the complementary advantages of the “structure–function” combined assessment: TCS can capture characteristic morphological changes, while TCCD reflects underlying cerebrovascular environmental abnormalities. Their combination provides a more comprehensive and multi-dimensional information perspective for PD diagnosis. This is particularly relevant in clinical practice, where a negative or inconclusive SN+ result does not reliably exclude PD; in such cases, the addition of MCA Vd assessment can help identify patients who might otherwise be missed by structural imaging alone.

Currently, most studies adopt a standard using an SN+ area ≥0.20 cm^2^ as the cut-off value (29). However, the cut-off value in this study was 0.24 cm^2^, which is slightly higher than the optimal thresholds reported in Western populations but aligns more closely with findings from Chinese cohorts (44). Comprehensively considered, this discrepancy may be attributed to anatomical differences between Chinese and European populations, such as reduced cranial length and height, resulting in a more rounded morphology.

Additionally, although the MCA Vd exhibits lower diagnostic specificity for PD compared with SN+, it may still serve as a valuable auxiliary indicator, as it could reflect the underlying cerebrovascular or autonomic dysfunction commonly observed in PD. Similar to the advantage of integrating motor and non-motor symptoms in PD assessment (45), the combination of structural and functional ultrasound parameters may provide a more comprehensive evaluation of the disease.

To further account for potential confounding by demographic factors, we performed a sensitivity analysis using ANCOVA with age and sex as covariates, which confirmed the robustness of our primary findings (see Methods). Nevertheless, we acknowledge that a formal covariate-adjusted ROC analysis was not performed, primarily owing to the exploratory nature of this study and the relatively limited sample size. Future studies with larger cohorts could employ this approach, such as calculating the area under the covariate-adjusted ROC curve, to more rigorously assess the independent contribution of the combined model beyond established clinical variables.

### 4.4 Overall contribution and clinical significance

Taken together, the present study demonstrates that integrating structural (SN+) and functional (MCA Vd) transcranial ultrasound parameters yields a diagnostic model with high sensitivity and markedly improved specificity (AUC = 0.945), outperforming either parameter alone. Beyond the statistical improvement, this “structure–function” framework offers a clinically practical, non-invasive strategy that can be readily implemented at the bedside or in outpatient settings. It reduces reliance on a single structural marker, which is particularly valuable when SN+ is equivocal or absent, and broadens the diagnostic perspective by capturing both nigral pathology and coexisting cerebrovascular dysfunction. In this sense, the combined assessment not only enhances diagnostic accuracy but also aligns with the emerging view that PD involves both neurodegenerative and vascular/autonomic components. However, these conclusions remain preliminary and must be interpreted in light of the study’s limitations.

### 4.5 Limitations and future prospects

This study has several limitations that should be acknowledged. First, the sample size was relatively limited—particularly for the PPS group—and the data were derived from a single center. Although 10-fold cross-validation was used for internal validation, the generalizability of our findings to other populations remains to be established. Future multi-center studies with larger cohorts, especially encompassing sufficient numbers of PPS subtypes, are warranted.

Second, we did not perform subtyping of Parkinson-plus syndromes (e.g., progressive supranuclear palsy, multiple system atrophy), owing largely to the small number of cases within each subtype. Consequently, the precise utility of transcranial ultrasound in differentiating PD from specific PPS subtypes could not be determined. This will be an important direction for future collaborative research.

Third, although UPDRS III scores and Hoehn & Yahr stages were collected for both groups, these disease severity measures were not incorporated into the diagnostic model, as the primary aim of this study was to evaluate the ultrasound parameters themselves. The relationship between ultrasound findings and clinical severity is being explored in a separate analysis.

Fourth, LEDD was significantly higher in the PD group than in the PPS group. Although LEDD was recorded and compared, its potential confounding effect on cerebral hemodynamic parameters could not be fully excluded, given the known vasodilatory properties of dopaminergic medications. Future prospective studies with strictly standardized medication status and LEDD included as a formal covariate are needed to clarify the independent contribution of ultrasound parameters.

Fifth, the measurement of substantia nigra hyperechogenicity by TCS relies on operator-based subjective delineation, which may introduce variability. Future work should incorporate computer-assisted quantitative analysis techniques to improve objectivity and reproducibility. In addition, the combined diagnostic model was developed within a single cohort without external validation; independent external cohorts are therefore required to confirm its robustness.

Finally, the diagnostic model was based on a simple logistic regression. More advanced machine learning approaches may further optimize diagnostic accuracy and reveal complex interactions among variables. Moreover, given the exploratory nature of our analyses and the multiple statistical comparisons performed across ultrasound parameters, the reported *p*-values should be interpreted with caution. Future confirmatory studies with pre-specified endpoints and appropriate multiplicity adjustments are needed to validate these findings.

Despite these limitations, the core finding—that combining TCS and TCCD parameters significantly improves diagnostic efficacy for PD—remains robust and clinically meaningful. Future large-scale, multi-center studies with rigorous PPS subtyping and computer-assisted quantitative analysis will help validate and extend our conclusions, promoting the standardization and precision of transcranial ultrasound in the differential diagnosis of parkinsonism.

## 5 Conclusion

In summary, this study demonstrates that a combined “structure–function” ultrasound model—integrating TCS-derived SN+ area with TCCD-derived MCA Vd—provides superior diagnostic accuracy for differentiating PD from PPS (AUC = 0.945) compared with either modality alone. This multimodal transcranial ultrasound approach offers a non-invasive, clinically feasible strategy to improve diagnostic precision, particularly in patients with equivocal or negative SN+ findings. More importantly, the inclusion of hemodynamic parameters highlights a valuable functional dimension beyond structural assessment, suggesting a novel direction for future PD diagnostic research. Future multi-center studies with external validation are warranted to confirm its generalizability and facilitate clinical translation.

## Data Availability

The data supporting the findings are included in the article and its Supplementary material. Further inquiries can be directed to the corresponding author.
