# Supplementary material for: The clinical value of combined assessment of substantia nigra morphology and intracranial hemodynamics via transcranial ultrasound in the diagnosis and differentiation of Parkinson’s disease
Source: Front Neurol. 2026 Sep 24;17:1913166. doi: 10.3389/fneur.2026.1913166 (PMC13645683; doi:10.3389/fneur.2026.1913166)
Supplement: Supplementary file 1 [file Table_1.docx]

Supplementary Table S1 Adjusted means and 95% confidence intervals for substantia nigra and hemodynamic parameters after controlling for age and sex (ANCOVA)

| Parameter | Group | Adjusted Mean | 95% CI | Adjusted P value |
| --- | --- | --- | --- | --- |
| Unilateral SN+ area (cm²) | PD | 0.26 | 0.24，0.28 | <0.001 |
|  | PPS | 0.15 | 0.12，0.18 |  |
|  | NC | 0.07 | 0.05，0.08 |  |
| Bilateral SN+ total area (cm²) | PD | 0.55 | 0.54，0.56 | <0.001 |
|  | PPS | 0.20 | 0.19，0.21 |  |
|  | NC | 0.15 | 0.14，0.16 |  |
| S/M ratio (%) | PD | 11.56 | 11.36，11.76 | <0.001 |
|  | PPS | 4.20 | 3.88，4.53 |  |
|  | NC | 3.10 | 2.91，3.29 |  |
| MCA Vd (cm/s) | PD | 35.04 | 34.27，35.82 | <0.001 |
|  | PPS | 36.07 | 34.82，37.31 |  |
|  | NC | 38.95 | 38.21，39.68 |  |
| BA PI | PD | 0.92 | 0.86，0.98 | <0.001 |
|  | PPS | 0.88 | 0.82，0.94 |  |
|  | NC | 0.69 | 0.63，0.75 |  |

Note: Adjusted means were estimated from ANCOVA with age and sex as covariates. Only parameters with statistically significant adjusted group differences (as reported in the main text) are shown. For parameters without significant differences after ANCOVA, the raw data are presented in Table 3 of the main text. Full ANCOVA results for all parameters are available upon request.
